# OLFM4 promotes the progression of intestinal metaplasia through activation of the MYH9/GSK3β/β-catenin pathway

**DOI:** 10.1186/s12943-024-02016-9

**Published:** 2024-06-07

**Authors:** Hongfa Wei, Wenchao Li, Leli Zeng, Ni Ding, Kuan Li, Hong Yu, Fei Jiang, Haofan Yin, Yu Xia, Cuncan Deng, Nan Cai, Xiancong Chen, Liang Gu, Huanjie Chen, Feiran Zhang, Yulong He, Jia Li, Changhua Zhang

**Affiliations:** 1 Department of General Surgery, The First Affiliated Hospital of Shantou University Medical College, Jinping, Shantou, Guangdong 515041 P.R. China; 2 Department of Gastrointestinal Surgery, Digestive Diseases Center, The Seventh Affiliated Hospital, Sun Yat-Sen University, Shenzhen, 518107 China; 3 Scientific Research Center, The Biobank, The Seventh Affiliated Hospital of Sun Yat-Sen University, Shenzhen, 518107 Guangdong P.R. China; 4 The Department of Thyroid and Breast Surgery, The Third Affiliated Hospital of Sun Yat-Sen University, Guangzhou, China; 5 Department of Laboratory Medicine, Shenzhen People’s Hospital, (The Second Clinical Medical College, Jinan University, The First Affiliated Hospital, Southern University of Science and Technology), Shenzhen, Guangdong China

## Abstract

**Background:**

Intestinal metaplasia (IM) is classified into complete intestinal metaplasia (CIM) and incomplete intestinal metaplasia (IIM). Patients diagnosed with IIM face an elevated susceptibility to the development of gastric cancer, underscoring the critical need for early screening measures. In addition to the complexities associated with diagnosis, the exact mechanisms driving the progression of gastric cancer in IIM patients remain poorly understood. OLFM4 is overexpressed in several types of tumors, including colorectal, gastric, pancreatic, and ovarian cancers, and its expression has been associated with tumor progression.

**Methods:**

In this study, we used pathological sections from two clinical centers, biopsies of IM tissues, precancerous lesions of gastric cancer (PLGC) cell models, animal models, and organoids to explore the role of OLFM4 in IIM.

**Results:**

Our results show that OLFM4 expression is highly increased in IIM, with superior diagnostic accuracy of IIM when compared to CDX2 and MUC2. OLFM4, along with MYH9, was overexpressed in IM organoids and PLGC animal models. Furthermore, OLFM4, in combination with Myosin heavy chain 9 (MYH9), accelerated the ubiquitination of GSK3β and resulted in increased β-catenin levels through the Wnt signaling pathway, promoting the proliferation and invasion abilities of PLGC cells.

**Conclusions:**

OLFM4 represents a novel biomarker for IIM and could be utilized as an important auxiliary means to delimit the key population for early gastric cancer screening. Finally, our study identifies cell signaling pathways involved in the progression of IM.

**Supplementary Information:**

The online version contains supplementary material available at 10.1186/s12943-024-02016-9.

## Introduction

Gastric cancer (GC) is a significant health concern, ranking fifth and fourth in terms of morbidity and mortality [1]. Early diagnosis rates for gastric cancer worldwide are currently below 20%, considerably lower than the 50%-60% rates seen in Japan and South Korea. This is primarily due to the hidden onset of gastric cancer, and the substantial cost of intensive early screening, which is not currently feasible on a widespread scale [2–6]. Targeted surveillance of precancerous lesions, including them in the high-risk group for critical monitoring, can improve the efficiency and financial benefits of screening for early gastric cancer (EGC). Chronic stimulation of normal gastric mucosa by factors like Helicobacter pylori (HP), carcinogens, high salt, bile acids, tobacco, or alcohol can lead to pathological progression from chronic atrophic gastritis (CAG), intestinal metaplasia (IM), gastric dysplasia, and ultimately adenocarcinoma, a process known as the "Correa cascade" [7, 8]. Intestinal metaplasia represents a significant portion of precancerous lesions associated with gastric cancer. Based on Lauren's classification, approximately 80% of gastric cancer cases are categorized as the intestinal type, which originates from the mucosa undergoing intestinal metaplasia [9–11]. Intestinal metaplasia results from chronic inflammatory stimulation of the stomach's normal mucosal epithelium, leading to atrophy of parietal cells and the formation of goblet cells and enterocytes. While enteroid epithelium replaces the lost gastric glands, this process is marked by impaired differentiation and atypical regeneration, increasing the risk of cancer [8, 12–14]. Consequently, patients with intestinal metaplasia have a significantly higher risk of developing cancer compared to healthy individuals [13, 15].

Incomplete intestinal metaplasia (IIM) is associated with a 4 to 11-fold higher risk of developing gastric adenocarcinoma compared to Complete intestinal metaplasia (CIM) [16–18]. Since various subtypes of intestinal metaplasia exhibit different levels of cancer risk, recognizing IIM is essential. Currently, HE staining and AB-PAS staining are the primary methods used for assessing IIM. However, staining interpretation can be challenging for non-pathologists as well as non-gastrointestinal pathological experts [13]. Therefore, developing appropriate markers for IIM diagnosis, identifying "high-risk" IIM groups, and defining key screening targets can enhance endoscopy efficiency, increase EGC diagnosis rates, and significantly reduce associated healthcare costs.

OLFM4, also known as Olfactomedin-4, GW112, or hGC-1, is a glycoprotein belonging to the olfactory regulatory protein family [19]. OLFM4 expression is absent in normal gastric mucosa but is present in the small intestine and colon [20, 21]. Researches have highlighted OLFM4's crucial role in regulating intestinal stem cells [22, 23]. In fact, one of OLFM4's key biological functions is to regulate cell adhesion and cell migration by interacting with adhesion molecules, the cytoskeleton, and the extracellular matrix [24]. Studies have shown elevated OLFM4 expression in gastric cancer and colorectal cancer, particularly in the early stages of tumor formation [25]. However, previous studies have not explored the differential expression of OLFM4 in different severity of intestinal metaplasia and the mechanism by which OLFM4 promotes the progression of intestinal metaplasia. The activation of the Wnt/β-catenin signaling pathway is crucial for tumor invasion and metastasis [26–28]. Phenotypically, Wnt/β-catenin expression is positive in 86% of intestinal metaplasia and 95% of gastric cancer cases [29].

Expanding upon the aforementioned literature review, our research study centered on evaluating OLFM4 expression in IIM. We aimed to construct a predictive model utilizing OLFM4 as a classifier for distinguishing different types of intestinal metaplasia. Additionally, we investigated potential mechanisms and activated signaling pathways by which OLFM4 contributes to the progression of gastric mucosal intestinal metaplasia.

### Methods and materials

#### Patient samples and cell lines

This study received approval from the Medical Ethical Committee of the Seventh Affiliated Hospital of Sun Yat-sen University and the People's Hospital of Fengqing in Yunnan Province (KY-2021–105-01, 2021/12/02). Written informed consent, following the Declaration of Helsinki guidelines, was obtained from every patient. Paraffin-embedded archived samples were collected between 2018 and 2022 from two distinct clinical centers. The samples of the first clinical center included 78 newly diagnosed intestinal metaplasia patients and 40 healthy individuals from the Seventh Affiliated Hospital (Training set). The samples of the second clinical center consisted of 63 newly diagnosed intestinal metaplasia patients and 40 healthy individuals from the People's Hospital of Fengqing (Validation set). The supplemental materials include the clinical data of the Training set and Validation set. Histological diagnoses were based on the Third edition of Pathology [30], and the classification of intestinal metaplasia was determined according to Pathology and classical literature [30–33]. GES-1 cells were purchased from ATCC and underwent short tandem repeat (STR) analysis.

Patient-derived organoids (PDO) were cultured in the laboratory of the Seventh Affiliated Hospital. Biopsy tissues were obtained from patients with intestinal metaplasia or healthy individuals. First, we obtained biopsy samples from the gastric mucosa suspected of intestinal metaplasia and digested them into generate organoids. Subsequently, based on postoperative pathology, we selected cases showing severe intestinal epithelial metaplasia to establish organoid models mimicking intestinal epithelial metaplasia. The collected gastric mucosal tissues were washed three times in penicillin–streptomycin mixed solution in PBS, then under aseptic conditions, cut into 1mm^3^ fragments. These fragments were subsequently incubated in DMEM/F12 medium with collagenase IV at 37 °C with agitation for 30 min. After centrifugation, the supernatant containing single-cell suspension was collected following removal of the sediment. The cell pellet obtained after centrifugation was resuspended in a mixture of DMEM/F12 and Matrigel matrix gel solution, and seeded into 96-well plates at 10μL per well. The gastric mucosal organoids were then incubated at 37 °C until the gel solidified, followed by addition of 200μL organoid culture medium containing penicillin–streptomycin mixture. The cultures were maintained at 37 °C with medium renewal every 3 days for 14 days. We adapted a protocol for organoid culture medium from Helen H.N. Yan, incorporating components such as DMEM/F12, HEPES, GlutaMax, penicillin/streptomycin, RSPO-1, B27, N-Acetylcysteine, EGF, FGF10, Noggin, A8301, Y-27632, and Gastrin into the culture medium [34].

#### DEGs in intestinal metaplasia tissues

We selected the GSE78523 dataset from the Gene Expression Omnibus database (GEO), which comprises samples from both normal gastric mucosa and intestinal metaplasia tissues. Differential Expression Genes (DEGs) were analyzed using the "DESeq2" package in R. The volcano plots and heatmaps were generated using the "ggplot2" package or GraphPad Prism 8.0.2 based on the results of the DEGs analysis.

#### Identification of a new PLGC subgroup in intestinal metaplasia tissues

We selected and analyzed the GSE134520 dataset, a single-cell RNA sequencing (scRNA-seq) dataset that includes non-atrophic gastritis (NAG), intestinal metaplasia (IM), and early gastric cancer (EGC), using the "Seurat" R package. Data normalization was performed using the "NormalizeData" function, and inconsequential sources of variation were removed with the "ScaleData" function. The "FindVariableFeatures" function was used to identify highly variable genes (HVGs), and the "RunPCA" function identified 50 significant principal component analyses (PCA). We embedded cells into the graph structure of PCAs using the "FindNeighbors" and "FindClusters" functions. The spatial correlation of expression data was presented through Uniform Manifold Approximation and Projection (UMAP) plots based on RunUMAP and Dimplot. We selected all epithelial cells using classical epithelial markers "EPCAM" and "KRT19". A total of 11 clusters were identified based on HVGs. To assess the malignancy of glandular cells, the "inferCNV" package was used to determine cellular heterogeneity by identifying chromosome copy number variation (CNV) in scRNA-seq. Gastric cancer cells served as a positive control for CNV, and precancerous lesions of gastric carcinoma (PLGC) cells were identified by "inferCNV" in comparison. The "Monocle" function was employed to display the evolution of gastric mucosa during EGC development by performing pseudotime analysis, projecting high-dimensional data into one dimension. The "Cytotrace" function was utilized to create a critical RNA-based feature for developmental potential and to establish a platform for delineating cellular hierarchies, attempting to predict differentiation states from scRNA-seq.

#### Reagents

N-Methyl-N”-nitro-N-nitrosoguanidine (MNNG) was obtained from Meilunbio (MB0455-2, China).

#### Cell transfection

Lentivirus vectors encoding shOLFM4, oeOLFM4, shMYH9, oeMYH9, control, or HA-ubiquitin plasmids were from GeneCopoeia (Guangzhou, China). HA-ubiquitin plasmids were transfected into cells for 48 h, followed by lysis for immunoblotting with anti-HA antibodies.

24 h prior to lentivirus transfection, adhere cells were seeded at a density of 1 × 10^5^ cells per well in a 24-well plate. 5 ug/ml of polybrene was added as a membrane-disrupting agent, followed by the establishment of five different virus concentration gradients (0, 1, 2, 3, 4, 5 ul/mL) and incubation at 37 °C. After 6 h, the media was replaced, and cells were further cultured until 48 h. Fluorescence expression was observed, and an appropriate virus concentration was selected for experimentation. Cell selection was performed using the purine resistance gene contained within the lentivirus. The sequence of plasmids as follows:

**Table:** 

	ID	Target Sequence
NC	CSHCTR001-LVRU6GP	GCTTCGCGCCGTAGTCTTA
EC	EX-NEG-Lv201	details in Supplementary Information
OLFM4	EX-Y2060-Lv201	details in Supplementary Information
shOLFM4-1	HSH090725-LVRU6GP-a	CCAAAGTGAGGGAATATGTCC
shOLFM4-2	HSH090725-LVRU6GP-b	CCTAACTGTCCGAATTGACAT
MYH9	EX-T1335-Lv242	details in Supplementary Information
shMYH9	HSH102697-LVRU6GP-a	GCAAGCTGCCGATAAGTATCT

#### Immunohistochemistry (IHC), Immunofluorescence (IF) staining, Alcian blue-periodic acid-Schiff (AB-PAS) straining, and hematoxylin–eosin (HE) staining

We conducted IHC, IF, AB-PAS, and HE staining using standard protocols as follows:

#### HE Staining

Deparaffinization of histopathological tissue paraffin sections and organoid cell paraffin sections in xylene followed by rehydration in distilled water. Sequential staining with hematoxylin, differentiation in 95% ethanol, eosin staining, dehydration in xylene, and mounting.

#### AB-PAS (Alcian Blue Periodic Acid Schiff) staining

Deparaffinization of histopathological tissue paraffin sections and organoid cell paraffin sections in xylene followed by rehydration in distilled water. Sequential staining with Alcian Blue, differentiation in 95% ethanol, eosin staining, PAS staining, dehydration in xylene, and mounting.

#### IHC or IF staining

Deparaffinization of histopathological tissue paraffin sections and organoid cell paraffin sections in xylene followed by rehydration in distilled water. High-temperature antigen retrieval with sodium citrate buffer at 121° C for 4 min, permeabilization with 3% H2O2, blocking of nonspecific binding sites with goat serum, overnight incubation at 4 °C with primary antibodies diluted accordingly, incubation at room temperature for 60 min with corresponding secondary antibodies (utilizing a universal mouse/rabbit secondary antibody labeled with horseradish peroxidase for IHC or Alexa Fluor 488/Alexa Fluor 647 fluorescent secondary antibodies for IF). IHC sections were stained with DAB (3,3'-Diaminobenzidine hydrochloride, GK600705), dehydrated in xylene, and mounted. IF sections were counterstained with DAPI fluorescence, antifade reagent before mounting and observed either through fluorescence microscopy (Leica, DM6B) or confocal microscopy (ZEISS, LSM-880). Two independent observers, unaware of the patient's clinical information, evaluated the staining results at separate intervals. The IHC score was determined using Image J to calculate the proportion of stained areas. Primary antibodies used included OLFM4 (14369S, CST,1:600), CDX2 (A19030, Abclonal, 1:800), MUC2 (sc-515032, Santa Cruz Biotechnology, 1:1000), MYH9 (14844–1-AP, Proteintech, 1:400), E-cadherin (60335–1-Ig, Proteintech, 1:200), Vimentin (60330–1-Ig, Proteintech, 1:100), and Ki67 (ab16667, Abcam, 1:1000).

#### Western Blot

Western Blot were performed following standard methods. Electrophoresis: Prepare Tris–Glycine gel and electrophoresis buffer. Load protein samples onto the lanes and set the voltage for electrophoresis (90 V for 30 min followed by 120 V for 90 min). Transfer: Soak PVDF membrane in methanol and then place it in transfer buffer. Place the membrane and gel in the transfer clamp, remove any bubbles, and proceed with the transfer (300 mA for 90 min). Block with 5% BSA for 60 min, incubate with the primary antibody overnight at 4℃, incubate with the secondary antibody for 60 min at room temperature, and expose using a Bio-Rad instrument after soaking in ECL developing solution. The primary antibodies included OLFM4 (14369S, CST, 1:1000), MYH9 (11128–1-AP, Proteintech, 1:5000), GSK3-β (22104–1-AP, Proteintech, 1:1000), β-catenin (51067–2-AP, Proteintech, 1:5000), β-actin (66009–1-Ig, Proteintech, 1:20000), p-STAT3 (9145S, CST, 1:2000), c-Myc (10828–1-AP, Proteintech, 1:2000), N-cadherin (22018–1-AP, Proteintech, 1:2000), E-cadherin (60335–1-Ig, Proteintech, 1:2000), Vimentin (60330–1-Ig, Proteintech, 1:20000), Snai1 (13099–1-AP, Proteintech, 1:500), Ubiquitin (10201–2-AP, Proteintech, 1:1000), and GAPDH (60004–1-Ig, Proteintech, 1:50000).

#### Quantitative Reverse Transcription-PCR (qRT-PCR)

Total RNA was harvested, and cDNA was generated by a reverse transcription reagent kit (AG11706, Accurate Biology, China). Then, the cDNA template was used for amplification with specific primers. qRT-PCR was conducted using SYBR-green PCR Master Mix and 45 cycles of 95℃ for 10 s, 60℃ for 20 s, and 72℃ for 20 s. These sequences of primers are defined as follows:

**Table:** 

	Forward	Reverse
CDX2	TTCACTACAGTCGCTACATCACCA	CTGCGGTTCTGAAACCAGATT
MUC1	TTCACCACCACCATGACACC	GGGGCTGTGGTAGCTGTAAG
MUC2	GGGGAGTGCTGTAAGAAGTGTGA	GTTGGAGACGGACGAGATGAG
OLFM4	GAGAAATCGTGGCTCTGAAGAC	CAGACGGTTTGCTGATGTTC
GSK3β	CATCCTTGGACTAAGGTCTTCCG	CATTTGTGGGGGTTGAAGCAG
β-actin	TCAAGATCATTGCTCCTCCTGAG	ACATCTGCTGGAAGGTGGACA

#### Cell proliferation assay, colony-formation assay, EdU assay, wound healing assay, and transwell assay

##### Cell proliferation

We assessed cell proliferation using the Cell Counting Kit-8 (CCK8) assay kit (Biosharp, China) and the Microplate Reader (BioTeK, USA). We seeded 2,000 cells into 96-well plates and cultured them for 1–5 days. Each day, we mixed the CCK8 reagent with the cell culture medium at a 1:9 ratio and incubated the cells for 90 min. We measured absorbance at 450 nm using a spectrophotometer in each culture dish.

#### Cell colony formation

For colony formation, we inoculated 800 cells in six-well plates and cultured them for 14 days. The number of cell colonies was determined by microscopy after staining with crystal violet dye.

#### Cell viability (EdU Assay)

We measured cell viability using the EdU assay. We plated 6,000 cells into 96-well plates, treated them with EdU reagent (10 μM, Beyotime, China), and observed them with fluorescence microscopy (Leica, DMI8).

#### Wound healing assay

Cells were plated and grown to confluence in six-well plates. We created scratches with a pipette tip and examined the cell migration process under a microscope at 0 and 24 h.

#### Cell migration and invasion

We evaluated cell migration and invasion using 24-well transwells (8.0 μm, Corning, USA), precoated with Matrigel in invasion assay but without Matrigel in migration assay. In the lower chamber, we added 500 μL RMPI-1640 with 10% FBS (Nanjing Ozfan). We seeded 5 × 10^4^ treated cells suspended in 500 μL RMPI-1640 without FBS in the upper chamber and cultured them at 37 °C for 36 h. We counted the number of GES-1 cells in the lower chamber using a cell counting plate.

#### Co-immunoprecipitation (IP) and mass spectrometry

Protein extraction and purification were performed using primary antibodies for IP and Protein A/G Magnetic Beads (B23202, Selleck). Mass spectrometry was performed by Baiqu Tech. co. LTD (Hangzhou, China) and results were provided in Table 5. The primary antibodies included OLFM4 (14369S, CST), MYH9 (11128–1-AP, Proteintech), and GSK3-β (22104–1-AP, Proteintech).

#### Cycloheximide (CHX) chase assay

Three groups of GES-1 cells were incubated with 2 μM MG132 (HY-13259, MCE, USA) for a duration of 12 h prior to protein extraction, while another groups of cells remained untreated. After the treatment of 20 μg/mL CHX (C7698, Sigma-Aldrich) for different times, cells were harvested and prepared for Western Blot.

#### PLGC animal model

The Institutional Animal Care and Use Committee (IACUC) (TopBiotech Co., LTD., Shenzhen) approved the experimental methods and animal use and care protocols. We obtained twenty male Sprague Dawley (SD) rats five-week-old for each group from Gempharmatech company (Jiangsu, China).

We prepared an MNNG solution with a concentration of 170 µg/ml by dissolving MNNG in drinking water containing 5% alcohol. The rats received the MNNG solution by gavage every two days, with a regimen of one day of a normal diet and one day of fasting. This procedure continued for 24 weeks.

#### Statistical analysis

Statistical analyses were performed by using SPSS 22.0 (SPSS Inc., Chicago). Histogram Graphing was performed with GraphPad Prism 8.0.2 (GraphPad Software). Each in vitro experiment was repeated three times or more and experimental data were depicted as mean ± standard deviation (SD). Quantitative variables were analyzed using a Student t-test for Gaussian distribution and non-parametric tests for non-Gaussian distribution. Differences were considered statistically significant at *p* < 0.05 (*p < 0.05, **p < 0.01, ***p < 0.001).

## Results

### OLFM4 is remarkably differentially expressed in intestinal metaplasia tissue

**Fig. 1:**
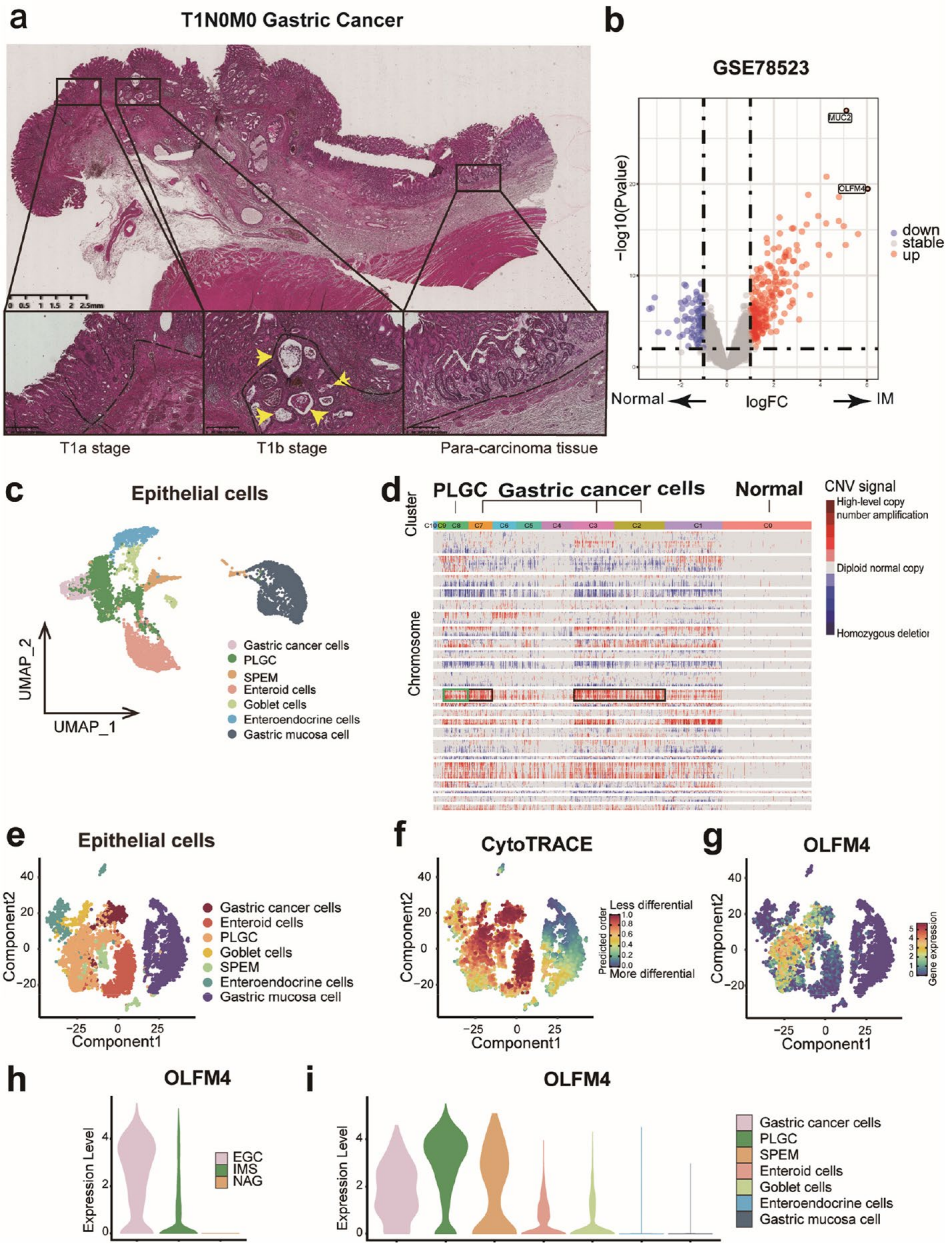
OLFM4 was the remarkable DEG in PLGC cells: **a** HE staining of pathological sections in T1N0M0 stage gastric cancer: Intestinal metaplasia lesions co-occurring with gastric cancer infiltration throughout the mucosal layer (left), infiltration that broke through the basement membrane and invaded the submucosa (middle), and normal adjacent glandular epithelium with accompanying intestinal metaplastic lesions (right). Key indicators: The Black dashed line represented the basement membrane, and the yellow arrow highlighted gastric cancer invading the basement membrane with intestinal metaplasia. **b** Volcano plot from GSE78523 highlighting the top two DEGs, OLFM4 and MUC2, in intestinal metaplasia tissues. **c** Cluster analysis of epithelial cells in GSE134520. **d** Analysis of CNV in epithelial cell subgroups using inferCNV. **e**–**g** Assessment of the differentiation of epithelial cell subgroups with Cytotrace. **h**-**i** Evaluation of OLFM4 expression in tissues and cell subgroups. IM: Intestinal metaplasia, EGC: Early gastric cancer, IMS: Sever intestinal metaplasia, NAG: non-atrophic gastritis, SPEM: Spasmolytic polypeptide expressing metaplasia, PLGC: Precancerous lesions of gastric carcinoma

Intestinal metaplasia tissues are closely associated with gastric cancer and are considered the origin of intestinal-type gastric cancer. In HE staining of pathological sections, the mucosal layer or submucosal layer of gastric cancer tissues, and para-cancerous tissues were observed to be accompanied by intestinal metaplasia lesions (Fig. 1a). To identify significant Differentially Expressed Genes (DEGs) in intestinal metaplasia tissue, we analyzed the RNA-seq dataset GSE78523, revealing high expression of OLFM4 and MUC2 in intestinal metaplasia tissue (Fig. 1b, Fig. S1a). As MUC2 is established as a conventional biomarker for intestinal metaplasia, our research endeavors have been directed towards exploring the role of OLFM4. Although it has been mentioned of OLFM4 among the highly expressed genes in intestinal metaplasia tissues by transcriptome sequencing, its expression profile and the mechanism by which it promotes progression in incomplete intestinal metaplasia remain unexplored [35, 36]. To further investigate OLFM4 in intestinal metaplasia, we analyzed the scRNA-seq dataset GSE134520. Traditional biomarkers, "EPCAM" and "KRT19", were used to distinguish epithelial cells and stromal cells, with epithelial cells constituting 88.4% of all cells (Fig. S1b-d). Epithelial cells were categorized into 11 clusters, yielding 7 cell subgroups based on their cell markers (Fig. 1c, Fig. S1e-f). To assess malignancy of epithelial cells, copy number variations (CNV) were analyzed in the 11 clusters using inferCNV (Fig. 1d). Clusters 2, 3, and 7 were identified as gastric cancer cells by their cancerous origin and frequent CNV (Fig. 1d, Fig. S1e). Cluster 8 was classified as PLGC cells based on their comparable frequency of CNV to gastric cancer cells and their partial cellular origin from intestinal metaplasia tissues (Fig. 1d, Fig. S1e). PLGC cells exhibited less differentiation across all subgroups and expressed high levels of OLFM4 (Fig. 1e-g) which was confirmed as a cell marker in PLGC cells (Fig. 1h-i, Fig. S1f-g). Pseudotime analyses indicated overlapping differentiation trajectories between PLGC cells and gastric cancer cells, suggesting a tendency toward malignancy in PLGC cells (Fig. 1f-g, Fig. S1h). Consequently, we speculated that OLFM4 might be one of the biomarkers in PLGC cells.

### OLFM4 is a novel biomarker of incomplete intestinal metaplasia

**Fig. 2:**
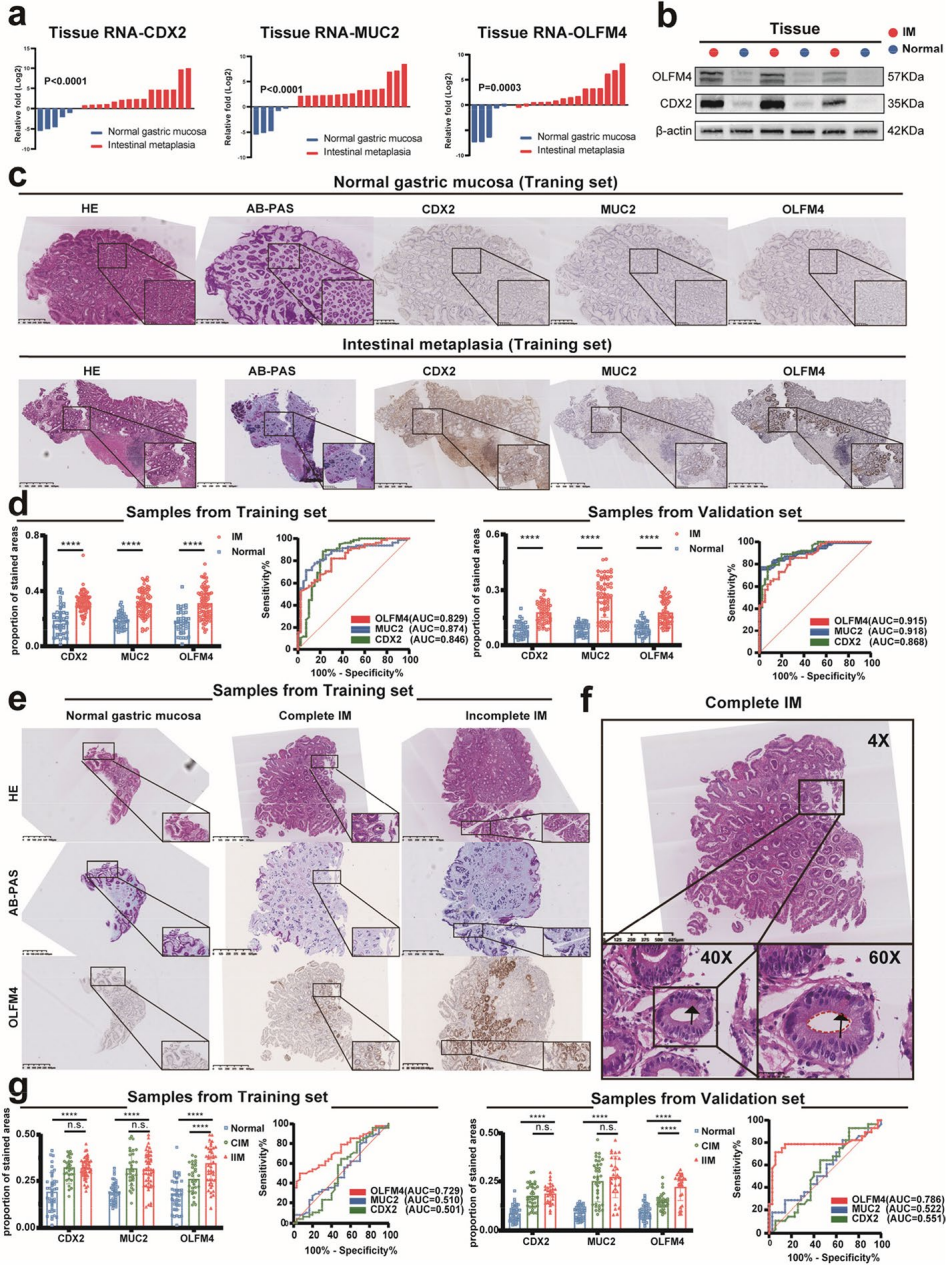
OLFM4 was the biomarker of IIM tissues: **a**, **b** qPCR and Western Blot were used to assess CDX2, MUC2, and OLFM4 expression in normal gastric mucosa and intestinal metaplasia tissues. **c** HE staining and AB-PAS staining confirmed intestinal metaplasia diagnosis and the immunohistochemical status of CDX2, MUC2, and OLFM4 was assessed in the Training set. **d** Statistical analysis of CDX2, MUC2, and OLFM4 immunohistochemical scores and diagnostic efficacy for intestinal metaplasia in both the Training set and the Validation set. **e** HE staining and AB-PAS staining confirmed intestinal metaplasia subtypes, while OLFM4 immunohistochemistry was used in the Training set. **f** HE staining of intestinal metaplasia demonstrated red-stained Paneth cells (black arrow) and an intact brush border (red dashed line), confirming CIM. (g) Statistical analysis of CDX2, MUC2, and OLFM4 immunohistochemical scores and diagnostic efficacy for IIM in both the Training set and Validation set. Statistics were expressed as mean ± SD. *p < 0.05, **p < 0.01, ***p < 0.001, ****p < 0.0001. N: Normal gastric mucosa; IM:Intestinal metaplasia

We selected IM biopsies and normal mucosa samples for qPCR and Western Blot analyses. Traditional biomarkers for IM, CDX2, and MUC2, exhibited significant increases in expression. Our target molecule, OLFM4, showed elevated expression in IM biopsies (Fig. 2a-b). To diagnose IM, we utilized HE staining and AB-PAS staining to assess the presence of Goblet cell in pathological sections of gastric lesions. Goblet cells are bright in HE staining, but dark blue in the AB-PAS staining (Fig. 2c, Fig. S2a). Statistical analysis revealed increased expression of CDX2, MUC2, and OLFM4 in IM tissues in both the Training set and Validation set (Fig. 2d). OLFM4 demonstrated an AUC value of 0.829 in the Training set and 0.868 in the Validation set (Fig. 2d, Table 1, Table 2). Certainly, the diagnostic effectiveness of OLFM4 in identifying IM was comparable to that of CDX2 and MUC2 (Fig. 2d).

**Table 1:** The ROC curve of IM in the Training set

Genes	Area	± SE	*P* value	95% CI		Youden index	
				**Up**	**Down**	**Sensitivity**	**Specificity**
CDX2	0.846	± 0.045	< 0.0001	0.759	0.934	88.5%	77.5%
MUC2	0.874	± 0.032	< 0.0001	0.812	0.936	70.5%	92.5%
OLFM4	0.829	± 0.038	< 0.0001	0.753	0.904	82.1%	70.0%

**Table 2:** The ROC curve of IM in the Validation set

Genes	Area	± SE	*P* value	95% CI		Youden index	
				**Up**	**Down**	**Sensitivity**	**Specificity**
CDX2	0.915	± 0.027	< 0.0001	0.862	0.968	77.8%	92.5%
MUC2	0.918	± 0.016	< 0.0001	0.877	0.938	76.2%	99.5%
OLFM4	0.868	± 0.034	< 0.0001	0.802	0.935	85.7%	72.5%

To distinguish between complete intestinal metaplasia (CIM) and incomplete intestinal metaplasia (IIM), we conducted HE and AB-PAS immunohistochemical experiments (Fig. 2e). HE staining revealed eosinophilic secretory granules in Paneth cells and intact brush borders in CIM tissues (Fig. 2f). In comparison to CIM, OLFM4 showed higher expression in IIM, while CDX2 and MUC2 displayed no significant differences (Fig. 2g, Fig. S2b). OLFM4 exhibited an AUC value of 0.729 in the Training set and 0.786 in the Validation set, indicating relatively better diagnostic performance compared to the other two biomarkers in IIM (Fig. 2g, Table 3, Table 4). Consequently, OLFM4 might serve as a superior biomarker for distinguishing IIM tissues in immunohistochemistry.

**Table 3:** The ROC curve of IIM in the Training set

Genes	Area	± SE	*P* value	95% CI		Youden index	
				**Up**	**Down**	**Sensitivity**	**Specificity**
CDX2	0.501	± 0.073	0.992	0.357	0.642	81.3%	19.2%
MUC2	0.510	± 0.068	0.886	0.376	0.644	87.5%	23.3%
OLFM4	0.729	± 0.056	0.001	0.620	0.838	52.0%	93.3%

**Table 4:** The ROC curve of IIM in the Validation set

Genes	Area	± SE	*P* value	95% CI		Youden index	
				**Up**	**Down**	**Sensitivity**	**Specificity**
CDX2	0.551	± 0.070	0.494	0.407	0.694	92.9%	28.6%
MUC2	0.522	± 0.074	0.485	0.407	0.696	82.1%	34.3%
OLFM4	0.786	± 0.069	< 0.0001	0.651	0.922	71.4%	94.3%

### Increased expression of OLFM4 in PLGC cells promotes cellular proliferation and invasion

**Fig. 3:**
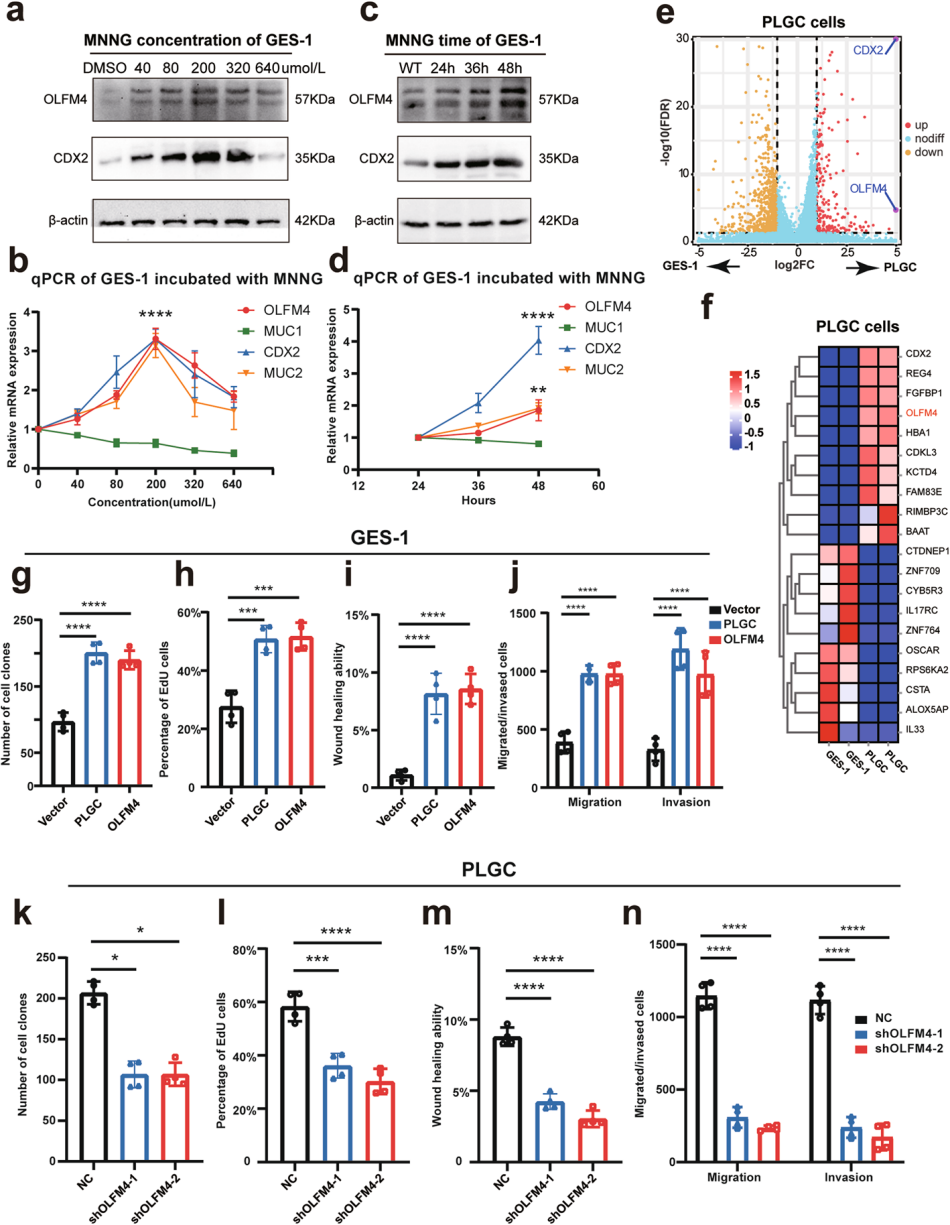
OLFM4 was a biomarker of PLGC cells: **a**-**d** qPCR and Western Blot were used to assess the markers of intestinal metaplasia in the GES-1 cells incubated with different concentrations or different times of MNNG. **e**-**f** Transcriptome sequencing of GES-1 cells induced with 200 μmol/L MNNG for 48 h had been conducted, the volcano plot and the heatmap were drawn to show the DEGs. **g**-**j** Statistical analysis of repeated experimental data for cell cloning, EdU, wound healing, and transwell assays in PLGC cells and GES-1 cells overexpressing OLFM4 (oeOLFM4). **k**-**n** Statistical analysis of repeated experimental data for cell cloning, EdU, wound healing, and transwell assays after OLFM4 knockdown in PLGC cells (shOLFM4). Statistics were expressed as mean ± SD. *p < 0.05, **p < 0.01, ***p < 0.001, ****p < 0.0001

To investigate the relationship between OLFM4 and intestinal metaplasia progression, we used a PLGC cell model by inducing GES-1 cells with MNNG exposure. The control group received PBS and the experimental group received MNNG dissolved in 0.3% DMSO to avoid cellular toxicity. As expected MNNG induced a significant increase in classical intestinal metaplasia biomarkers in PLGC cells, especially at a concentration of 200 μmol/L for 48 h (Fig. 3a-d). Subsequent transcriptome sequencing of PLGC cells confirmed markedly high expressions of CDX2 and OLFM4, validating the successful construction of MNNG-induced PLGC cells (Fig. 3e-f). Consistent with a role for OLFM4 in cancer cell proliferation, our experiments showed increased proliferation and enhanced invasion capabilities of both PLGC cells and OLFM4-overexpressing GES-1 cells (oeOLFM4) (Fig. 3g-j, Fig. S3a-f). Conversely, the knockdown of OLFM4 (shOLFM4) in PLGC cells led to decreased proliferation and invasion capabilities (Fig. 3k-n, Fig. S3g-l). These data confirmed the role of OLFM4 in PLGC progression.

### OLFM4 cooperates with MYH9 to activate the Wnt signaling pathway and enhance the progression of intestinal metaplasia

**Fig. 4:**
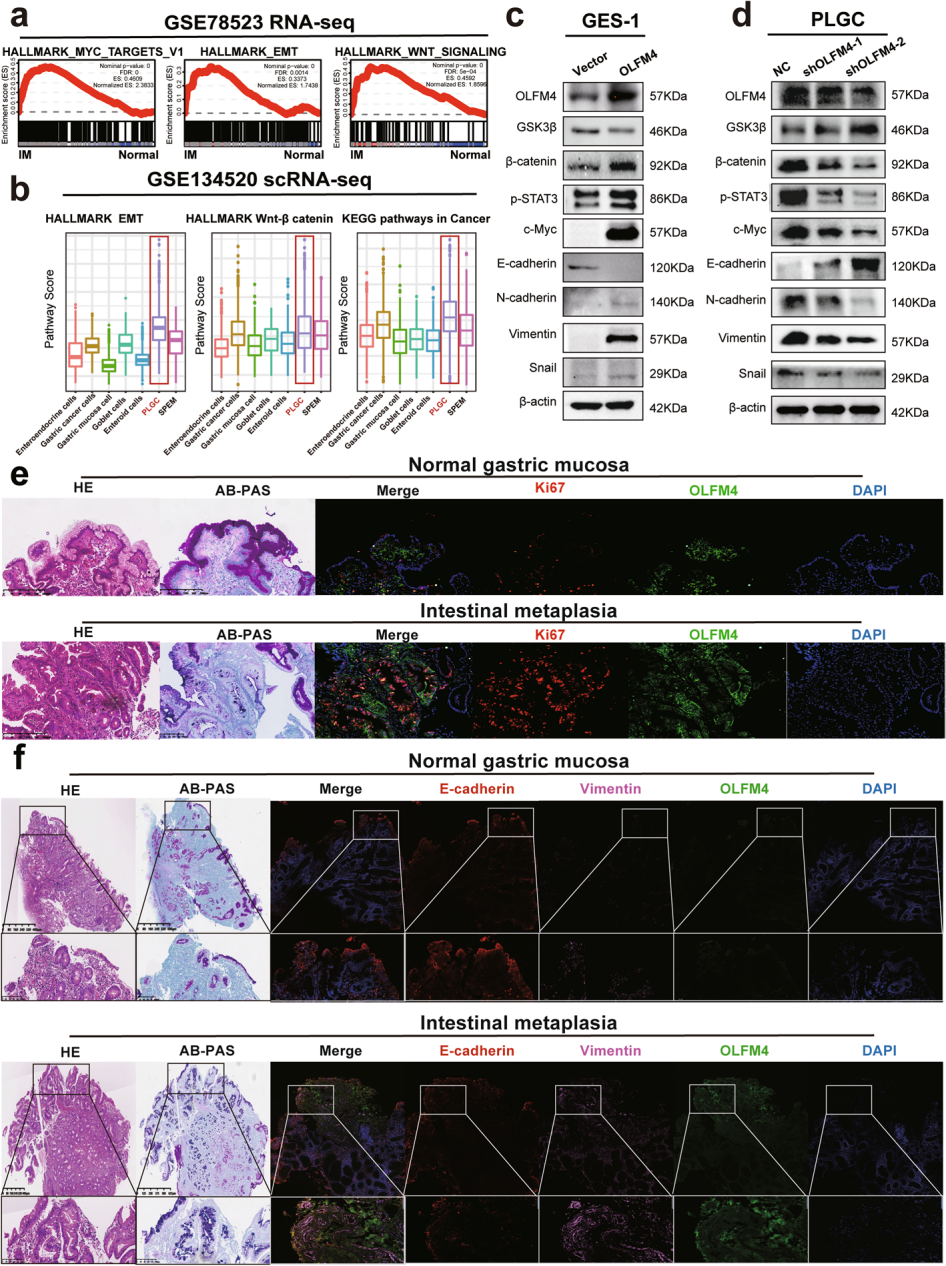
PLGC cells with high OLFM4 expression displayed activation of the Wnt signaling pathway: **a** The GSEA of the GSE78523 RNA-seq dataset in Hallmark genes and KEGG was analyzed. **b** The Addmodulescore analysis of the scRNA-seq dataset GSE134520 was analyzed to exhibit the functions enriched in the PLGC cells with high OLFM4 expression. **c**-**d** Western Blotting confirmed that the relation between the expression of OLFM4 and the expression of molecules related to Wnt/β-catenin signaling pathway, EMT transition, and c-Myc. **e**-**f** HE staining and AB-PAS staining were conducted to diagnose intestinal metaplasia, while the confocal immunofluorescence was performed to show the expression of OLFM4, Ki67, E-cadherin and Vimentin

We employed bioinformatics methods, specifically Gene Set Enrichment Analysis (GSEA) in Hallmark and KEGG, to analyze the RNA-seq dataset GSE78523 for mechanistic insights. High OLFM4 expression in intestinal metaplasia tissues was associated with enrichment in the Myc, EMT, and Wnt signaling pathways (Fig. 4a). Additionally, utilizing the AddModuleScore analysis on the scRNA-seq dataset GSE134520, we observed that PLGC cells with elevated OLFM4 expression exhibited the highest scores in Cancer, EMT, and Wnt/β-catenin signaling pathways (Fig. 4b). Western Blot assays confirmed a positive correlation between OLFM4 and Wnt pathway markers, EMT transition markers, c-Myc (Fig. 4c-d). Immunofluorescent staining confirmed an overlap expression of OLFM4, Ki67 and Vimentin in intestinal metaplasia tissues suggesting that OFLM4 stimulated proliferation and invasion (Fig. 4e-f).

**Fig. 5:**
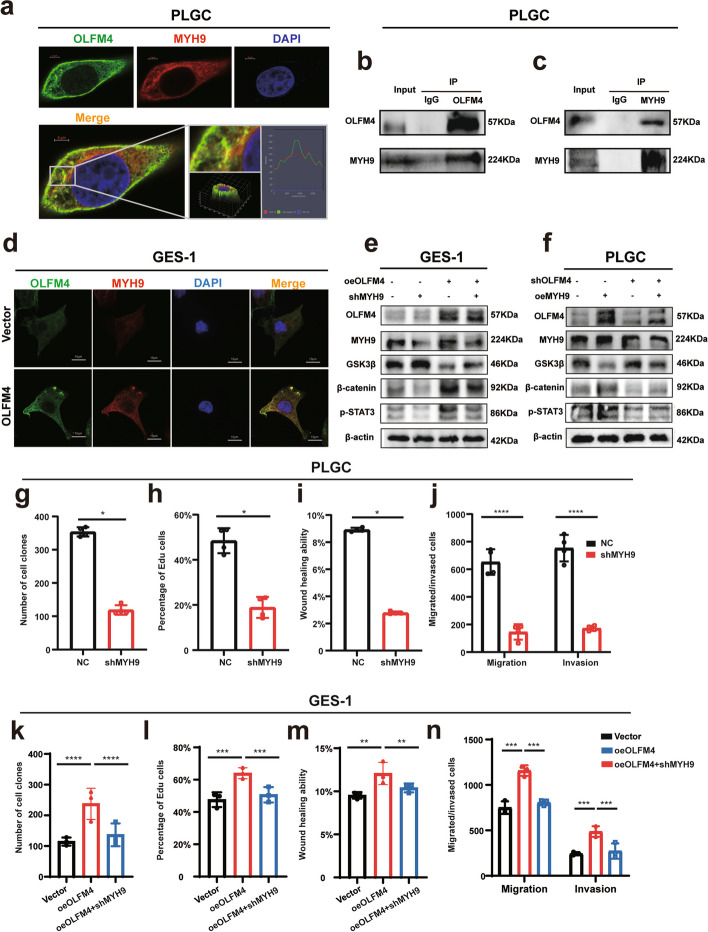
OLFM4 interaction with MYH9 stimulated activation of the Wnt pathway: **a** The figures demonstrated the co-localization of OLFM4 (green) and MYH9 (red) in fixed and immunostained cells. Cells were imaged using confocal microscopy, and the overlay image (yellow) indicated regions of co-localization, where both proteins were present in close proximity. Nuclei were stained with DAPI (blue) to provide cellular landmarks. The scale bar representeds 5 μm. **b**-**c** In the forward Co-IP, an antibody specific to OLFM4 was used to immunoprecipitate the protein complex from cell lysates. Western Blot analysis subsequently revealed the presence of MYH9 in the immunoprecipitated complex, indicating that MYH9 was binding with OLFM4. Conversely, in the reverse Co-IP, an antibody specific to MYH9 was employed to immunoprecipitate the associated proteins. Western Blot analysis confirmed the presence of OLFM4 in the immunoprecipitated complex. **d** After overexpressing OLFM4 in GES-1 cells, the expression level of MYH9 was evaluated through confocal immunofluorescence analysis. The scale bar representd 10 μm. **e**-**f** Western Blot analysis demonstrated the effects of overexpressing OLFM4 followed by knocking down MYH9, or overexpressing MYH9 followed by knocking down OLFM4 on the expression levels of Wnt pathway signals. **g**-**n** Statistical analysis of repeated experimental data for cell cloning, EdU, wound healing, and transwell assays after MYH9 knockdown in PLGC cells (shMYH9) or in oeOLFM4 cells. Statistics were expressed as mean ± SD. *p < 0.05, **p < 0.01, ***p < 0.001, ****p < 0.0001

To identify OLFM4-interacting proteins and associated signaling pathways involved in intestinal metaplasia, we conducted Co-immunoprecipitation (Co-IP) assays and mass spectrometry-based quantitative proteomics. Table 5 displayed the top five proteins and their interaction scores. Interestingly, MYH9 was a gene closely related to the progression and poor prognosis of gastric cancer and esophageal cancer, and ranked first. Confocal immunofluorescence and Co-IP assays confirmed endogenous MYH9 as a novel OLFM4-associated protein (Fig. 5a-c). In addition, MYH9 expression increased with OLFM4 overexpression in GES-1 cells (Fig. 5d). Furthermore, the expression of MYH9 was upregulated in pathological specimens of intestinal metaplasia tissues (Fig S4a). Western Blot assays suggested that the interaction between OLFM4 and MYH9 activate the Wnt signaling pathway (Fig. 5e-f). Furthermore, the knockdown of MYH9 in PLGC cells or in oeOLFM4 cells significantly weakened cellular proliferation and invasion abilities (Fig. 5g-n, Fig S4b-k).

**Table 5:** The top 5 proteins that binding to OLFM4 protein in mass spectrometry

ID	Gene	MW [kDa]	Score Sequest HT	Abundance	Protein Unique Peptides	Unique Peptides	Peptides (by Search Engine)	Wnt/β-catenin pathway
P35579	MYH9	226.4	250.94	1,306,113,914	55	60	70	[37, 38]
P35580	MYH10	229.0	183.77	228,611,562	39	43	53	[39, 40]
P60709	ACTB	41.7	98.44	2,971,294,207	0	3	14	[41]
P63267	ACTG2	41.9	79.64	4,936,448	0	3	27	[42]
P21333	FLNA	280.7	75.08	31,837,103	27	27	18	[43, 44]

### OLFM4 regulates the Wnt signaling pathway by influencing the ubiquitination of GSK3β in intestinal metaplasia

**Fig. 6:**
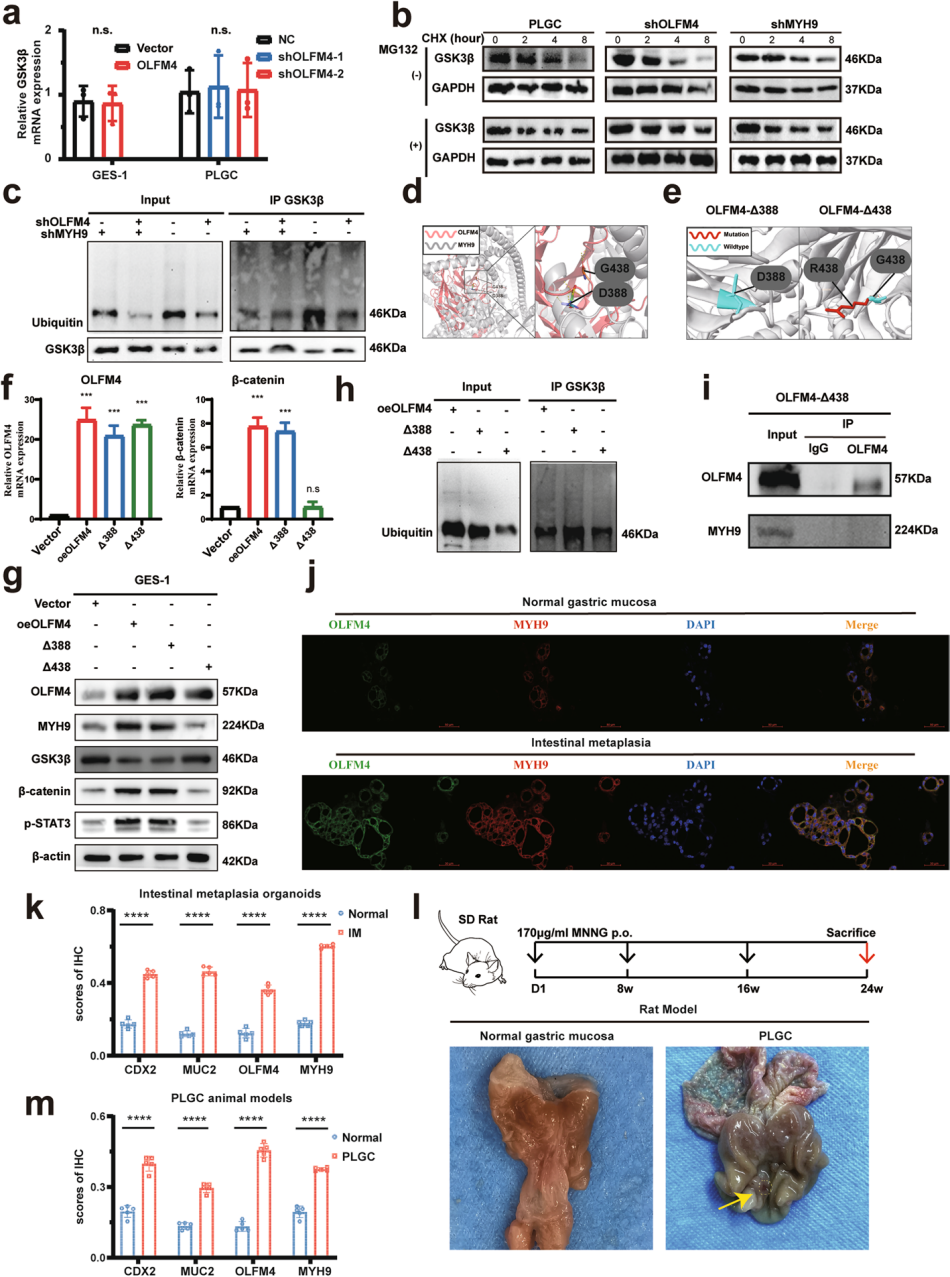
OLFM4 interacted with MYH9 to facilitate the ubiquitination of GSK3β: **a** qPCR assays demonstrated that relative GSK3β mRNA levels after GES-1 cells overexpression of OLFM4 or PLGC cells knockdown of OLFM4. **b** The CHX assay was performed by incubating the cells with CHX for different periods of time with or without MG132. Cycloheximide, CHX, an intracellular protein synthesis inhibitor. MG132, a proteasome inhibitor. **c** Western Blot experiments presented the effects of knocking down OLFM4 or MYH9 on ubiquitination levels and the expression of GSK3β in the Co-IP assay of PLGC cells. **d** The Pymol software constructed two crucial protein–protein dockings between OLFM4 and MYH9. **e** The Missense3D database predicted and constructed two mutations of OLFM4 protein. **f**-**g** GES-1 cells were transfected with vector, OLFM4 or OLFM4 mutation plasmids, and then subjected to qPCR and Western Blot for analysis the effect on the Wnt signaling pathway. **h** GES-1 cells were transfected with OLFM4 mutation plasmids and conducted with Co-IP assay for the ubiquitination level of GSK3β. **i** GES-1 cells were transfected with G438 mutation plasmids, and the Co-IP assay was executed to the interaction between OLFM4 and MYH9. **j** Immunofluorescence assays highlighted the elevated expression of OLFM4 and MYH9 in intestinal metaplasia organoids compared to normal gastric mucosa. **k** Statistical analysis of immunohistochemical scores for CDX2, MUC2, OLFM4, and MYH9 in intestinal metaplasia organoids. **l** Illustration of the PLGC animal model construction process and the presentation of rat gastric mucosa. **m** Statistical analysis of immunohistochemical scores of CDX2, MUC2, OLFM4, and MYH9 in the gastric mucosa of PLGC rats. Statistics were expressed as mean ± SD. *p < 0.05, **p < 0.01, ***p < 0.001, ****p < 0.0001

Previous studies have shown that GSK3β interact with MYH9 to regulate the Wnt pathway [38]. Our study revealed that an increase in OLFM4 protein expression was associated with a decrease in GSK3β protein expression. However, GSK3β mRNA levels remained unchanged (Fig. 6a), we analyzed the effects of OLFM4 on GSK3β protein turnover. Clearly, CHX assays demonstrated that knocking down of either OLFM4 or MYH9 extended the half-life of GSK3β in PLGC cells (Fig. 6b). GSK3β possesses ubiquitination function [45–47]. Co-IP assays revealed transient transfection of OLFM4 or MYH9 knockdown plasmids in PLGC cells increased the expression of GSK3β, which implicated enhancing the intracellular ubiquitination level, which elucidated the role of OLFM4 and MYH9 in the promotion between ubiquitin and GSK3β in PLGC cells (Fig. 6c). Eleven candidate structure-damaged variants of the OLFM4 protein with highest Polyphen prediction scores were predicted in Missense3D database (Supplemental Table 1). The Pymol software was used to analyze the protein–protein docking between OLFM4 and MYH9. Two localizations (D388 and G438) of OLFM4, which had polar contacts with MYH9, were screened out for further mutation experiments (Fig. 6d). The 3D structural changes of missense mutations were constructed and found that the G438 site could form a significant mutation (Fig. 6e). Our results showed that the G438 mutation reversed the Wnt signal pathway activation in oeOLFM4 cells (Fig. 6f-g). The G438 mutation couldn’t increase the ubiquitination level of GSK3β in oeOLFM4 cells (Fig. 6h). Further Co-IP assay showed that the G438 mutation of OLFM4 protein could not have interaction with MYH9 (Fig. 6i). Our studies suggest that OLFM4 could interact with MYH9 to facilitate the ubiquitination of GSK3β, consequently activating the Wnt signaling pathway.

### OLFM4 and MYH9 increase in intestinal metaplasia organoids and PLGC animal models

PDO experiments using both intestinal metaplasia and normal gastric mucosa organoids revealed significantly elevated expressions of CDX2 and MUC2 in the intestinal metaplasia organoids. The immunofluorescence and immunohistochemical assays demonstrated robust expression of OLFM4 and MYH9 in the intestinal metaplasia organoids (Fig. 6j, k, Fig. S5a). Further qPCR demonstrated the mRNA expression of CDX2, MUC2, OLFM4 was increased in intestinal metaplasia organoids (Fig. S5b). To investigate the growth kinetics of intestinal metaplasia organoids, we conducted a CCK8 assay and discovered that these organoids exhibited a superior growth capacity (Fig. S5c).

In the PLGC animal model induced by MNNG, the gastric mucosa exhibited deep, extensive ulcers surrounded by red and swollen tissues (Fig. 6l). Under light microscopy, the gastric mucosal glands of PLGC models in HE staining exhibited swelling and disorganization, with a significant increase in heterogeneous cells that were notably aggravated (Fig. S5d). Furthermore, the conventional biomarkers CDX2 and MUC2, alongside OLFM4 and MYH9, displayed heightened expression levels in the PLGC models (Fig. 6m, Fig. S5d).

**Fig. 7:**
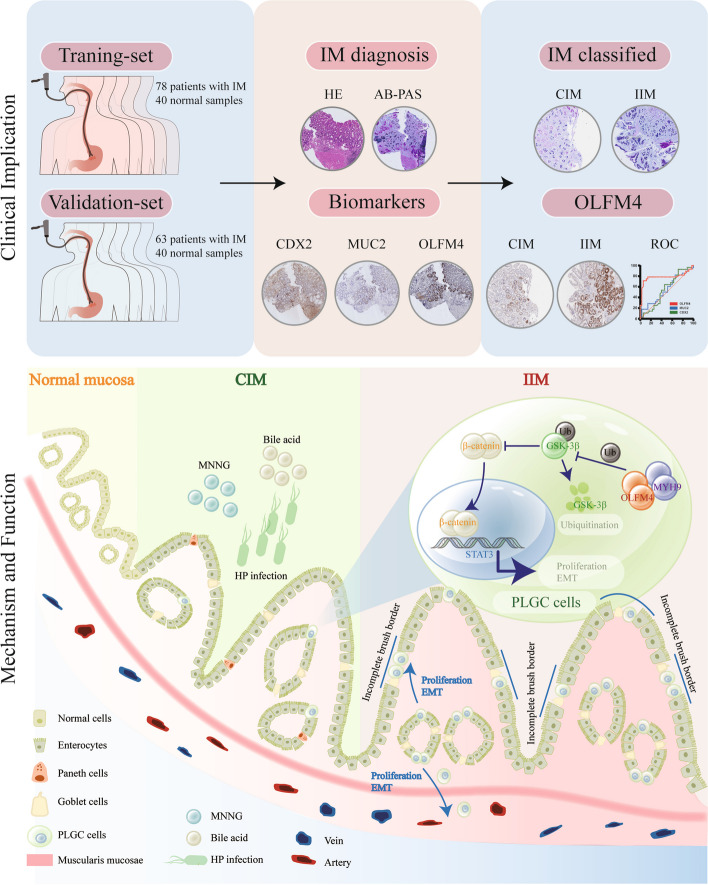
The study overview and the mechanism of OLFM4 promoting intestinal metaplasia: OLFM4 is highly expressed in incomplete intestinal metaplasia, and interacts with MYH9 to promote the progression of intestinal metaplasia via GSK3β/β-catenin signaling pathway

Overall, OLFM4 was the biomarker of IIM tissue and might promote the progression of intestinal metaplasia through the MYH9/GSK3β/β-catenin pathway (Fig. 7).

## Discussion

In this study, we identified OLFM4 as a novel biomarker of incomplete intestinal metaplasia (IIM) and discovered that OLFM4 promoted the progression of IIM through the MYH9/GSK3β/β-catenin pathway. These findings had significant implications for the development of novel biomarkers for IIM and novel therapeutic strategies for preventing the progression of intestinal metaplasia.

The annual incidence of gastric cancer in patients with intestinal metaplasia was 12.4/10000 (95%CI: 10.7–14.3), which was significantly higher than that in healthy people (2/100000–5/100000) [48]. A study from Sweden showed that 1 in 39 patients with intestinal metaplasia would develop gastric cancer within 20 years, a much higher incidence than in healthy people [49]. However, the prevalence of intestinal-type gastric cancer was greater in IIM, which had a 4 to 11fold higher risk of suffering gastric adenocarcinoma than CIM, and the risk rose with the severity of IIM [16–18]. In agreement with previous studies, we found that intestinal-type gastric cancers were frequently accompanied by incomplete intestinal metaplasia tissues suggesting that IIM was an important precancerous lesion in gastric cancer development. Unfortunately, while being good indicators for intestinal metaplasia, CDX2, and MUC2 could not be employed as biomarkers for IIM [50, 51]. This result was also validated by the pathology data from multi-center studies used in our investigation. Therefore, the identification of IIM lesions was of utmost importance in determining the high-priority population for early screening of gastric cancer. Nevertheless, the diagnosis of IIM posed significant challenges, and there were no better biomarkers specific to incomplete intestinal metaplasia tissues up to now. Furthermore, few investigations focused on the progression of intestinal metaplasia.

Studies had reported that the subtype of IM could be determined by CD10 staining of the brush surface of IM cells in the gastric mucosa, with a sensitivity of 87.5% and a specificity of 96.7% [52]. However, accurately identifying IIM based solely on sporadic incomplete brush border features was not feasible [33]. In HID-AB staining, the mucin profile in intestinal metaplasia cells could be used as a criterion when identifying the classification of intestinal metaplasia: mucin sulfate or salivary mucin in IIM cells could be stained brownish black by HID solution, while proteoglycans and hyaluronic acid in CIM cells were stained blue by Alcian solution. However, both HID-AB staining and AB-PAS staining methods were unable to provide clear insights into the stemness characteristics and proliferation of IIM. Additionally, the HID-AB staining method had toxicity concerns [53]. Therefore, the HID-AB staining had limited application in clinical practice. In the same study, it was suggested that Das1 staining could detect goblet cells in IIM tissues. However, goblet cells definitely did not constitute a prominent characteristic of IIM, and the Das1 staining method exhibited limited specificity and sensitivity. Consequently, it could not categorize intestinal metaplasia [52]. OLFM4 expression was closely related to cell stemness, reflecting the strong proliferative ability of cells with high OLFM4 expression [22, 54]. OLFM4 had been identified from a gene signature of Lgr5^+^ stem cells as a strong marker for murine small intestine stem cells [22]. The precancerous lesions in the stomach and esophagus exhibited significant levels of OLFM4 expression, which was positively correlated with the severity of diseases [25]. Here, we demonstrated that OLFM4 was a more effective biomarker for identifying IIM because it was expressed in intestinal metaplasia tissues, its detection was highly specific in IIM, and OLFM4 had higher diagnostic performance. Identifying and diagnosing IIM could play a crucial role in identifying individuals who should be prioritized for early screening of gastric cancer. This could significantly enhance the effectiveness of endoscopic screening and serve as a valuable auxiliary tool for early detection in screening programs.

The role of OLFM4, a gene associated with stemness properties, had received limited attention in the context of intestinal metaplasia. The amount of OLFM4^+^ cells was positively associated with the number of intestinal stem cells and the expression of OLFM4 was highly confined to the Lgr5^+^ stem cell area [22, 25, 54]. Some studies reported that OLFM4 could promote gastric cancer progression by promoting cell proliferation and invasion [55, 56]. In support of this, knockdown of OLFM4 expression greatly decreased cell growth and promoted apoptosis of gastric cancer cells [56–58]. Nonetheless, there was limited literature that specifically highlighted the elevated expression of OLFM4 in intestinal metaplasia tissues [25, 35]. OLFM4 expression was elevated in gastric and colorectal cancer, particularly in the early stages of tumor formation [25]. Other investigations had found that OLFM4 expression was higher in early-stage, moderately differentiated, and well-differentiated cancers, while it was considerably lower in late-stage, poorly differentiated, and undifferentiated tumors [59, 60]. OLFM4 expression was also associated with activation of the Wnt/β-catenin signaling pathway, which played a key role in regulating cell growth and differentiation [26–28, 61]. However, no literature explored the clinical application value, the biological function, and the mechanism of OLFM4 in intestinal metaplasia, especially in IIM. OLFM4 was significantly increased in IIM, possibly because OLFM4 matched the specific stemness characteristic of IIM [62]. Nevertheless, due to the absence of an extended follow-up period, it remained unclear whether IIM patients exhibiting high OLFM4 expression eventually developed gastric cancer. Collectively, our findings indicated that OLFM4 expression could serve as a pathologic predictor in IIM. MNNG, a chemical carcinogen, had been used to induce precancerous lesions of gastric cancer (PLGC) cells models and mimic PLGC in animal models [63–66]. Previous studies had shown that c-Ras, c-met, and ErbB2 mutations in PLGC cells caused GES-1 carcinogenesis in MNNG-induced PLGC cell models [67, 68]. Thus, the PLGC cells, induced by MNNG, was a widely recognized cell model of intestinal metaplasia [69–71]. Numerous animal experiments confirmed that Oral administration of MNNG in Sprague Dawley (SD) rats was associated with the development of PLGC [72, 73]. However, none of these experiments was supported by sequencing data. We performed transcriptome sequencing (RNA-seq) on PLGC cells, and discovered that they strongly expressed CDX2, making them an ideal cell model for researching intestinal metaplasia. Concurrently, our RNA-seq data confirmed the significant upregulation of OLFM4 in PLGC cells. In this study, we cultured intestinal metaplasia organoids which simulated and retained the information and functionality of intestinal metaplasia tissues [74]. The OLFM4 and MYH9 expression were both shown to be higher in intestinal metaplasia organoids than normal gastric mucosa, which was compatible with the PLGC cells model. Moreover, previous investigations had indicated that PLGC cells exhibited enhanced proliferative and invasive characteristics, consistent with our own observations from functional experiments conducted on PLGC cells [69–71].

In this study, we also demonstrated that OLFM4, combined with MYH9, was responsible for intestinal metaplasia progression. The overexpression of OLFM4 and the ability of malignant biological behavior including proliferation, migration and invasion were synchronously enhanced in PLGC cells. Mechanistic investigations revealed that OLFM4 played a role in activating the downstream signaling pathways of Wnt/β-catenin during the formation of intestinal metaplasia. This activation was involved in mediating tumor growth and the process of epithelial-mesenchymal transition (EMT). The Wnt pathway had been reported to be expressed in 86% of intestinal metaplasia, suggesting that Wnt played an important role in intestinal metaplasia [29]. Intriguingly, we demonstrated that MYH9 and OLFM4 cooperated to regulate the ubiquitination and degradation of GSK3β and to establish a positive regulatory loop MYH9/GSK3β/β-catenin involved in the progression of intestinal metaplasia. Myosin heavy chain 9 (MYH9) encoded non-muscle myosin II (NMM-II) played a crucial role in cell adhesion, migration, proliferation, and differentiation. Studies had found that MYH9 could promote the progression of liver cancer and lymphoma through the Wnt pathway [37, 38]. Considering that MYH9 overexpression had been associated with gastric cancer (GC) progression and unfavorable prognosis, it was noteworthy that MYH9 exhibited significant positive correlations with parameters such as depth of invasion (T stage), lymph node metastasis (N stage), distant metastasis (M stage), and the overall pTNM stage of gastric cancer [75]. MYH9 overexpression in gastric cancer cells had been shown to enhance their capacity for invasion and metastasis [76]. Prior scientific investigations had established that TUBB4A had the ability to bind with MYH9, facilitating the ubiquitination of GSK3β, thereby propelling the advancement of prostate cancer [45]. Furthermore, PTGDS engaged with MYH9 to ubiquitinate and degrade GSK3β, activating the Wnt/β-catenin signaling cascade, which exacerbated the malignancy of diffuse large B-cell lymphoma [37]. Additionally, SAMD9 interacted with MYH9, promoting the ubiquitination of GSK3β, leading to esophageal cancer recurrence due to β-catenin disinhibition [46]. Notably, ACTN1 enhanceds MYH9-induced GSK3β ubiquitination, promoting drug resistance and tumorigenesis in head and neck squamous cell carcinoma [47]. In the present study, #438 Gly site of OLFM4 was definitively identified as the interaction site with MYH9. The interaction between OLFM4 and MYH9 coordinately regulated the ubiquitination and degradation of GSK3β, ultimately releasing the inhibited β-catenin protein. This release led to an increase in β-catenin expression within the cytoplasm, followed by its translocation to the nucleus, where it participated in the regulation of downstream factors. Consequently, this cascade enhanced cellular proliferative and invasive capabilities, finding that align with previous research endeavors.

In summary, our results established, for the first time, the high expression and oncogenic significance of OLFM4 in intestinal metaplasia. Overall, our results showed that OLFM4 was a novel target for intestinal metaplasia treatment, and targeted inhibition of OLFM4 might be an effective treatment option for intestinal metaplasia.

### Supplementary Information

Supplementary Material 1.

Supplementary Material 2.

Supplementary Material 3.

## Data Availability

No datasets were generated or analysed during the current study.

## Abbreviations

IM: Intestinal metaplasia
CIM: Complete intestinal metaplasia
IIM: Incomplete intestinal metaplasia
PLGC: Precancerous lesions of gastric carcinoma
GC: Gastric cancer
EGC: Early gastric cancer
NAG: Non-atrophic gastritis
IMS: Sever intestinal metaplasia
SPEM: Spasmolytic polypeptide expressing metaplasia
HP: Helicobacter pylori
scRNA-seq: Single-cell RNA sequencing
CAG: Chronic atrophic gastritis
PDO: Patient-derived organoids
GEO: Gene Expression Omnibus database
DEGs: Differentially expressed genes
CNV: Chromosome copy number variation
HVGs: Highly variable genes
PCA: Principal component analyses
UMAP: Uniform Manifold Approximation and Projection
STR: Short tandem repeat
MNNG: N-Methyl-N”-nitro-N-nitrosoguanidine
SD rats: Sprague Dawley rats
IHC: Immunohistochemistry
IF: Immunofluorescence
HE: Hematoxylin–eosin
AB-PAS: Alcian blue-periodic acid-Schiff
qRT-PCR: Quantitative Reverse Transcription-PCR
CCK8: Cell Counting Kit-8
IP: Immunoprecipitation
CHX: Cycloheximide
AUC: Area under the curve
ROC: Receiver Operating Characteristic
GSEA: Gene Set Enrichment Analysis
EMT: Epithelial-mesenchymal transition
NMM-II: Non-muscle myosin II
IACUC: The Institutional Animal Care and Use Committee
