# Supplementary material for: OLFM4 promotes the progression of intestinal metaplasia through activation of the MYH9/GSK3β/β-catenin pathway
Source: Mol Cancer. 2024 Jun 7;23:124. doi: 10.1186/s12943-024-02016-9 (PMC11157765; doi:10.1186/s12943-024-02016-9)
Supplement: Supplementary file 1 — Supplementary Material 1. [file 12943_2024_2016_MOESM1_ESM.docx]

**Supplemental figures**


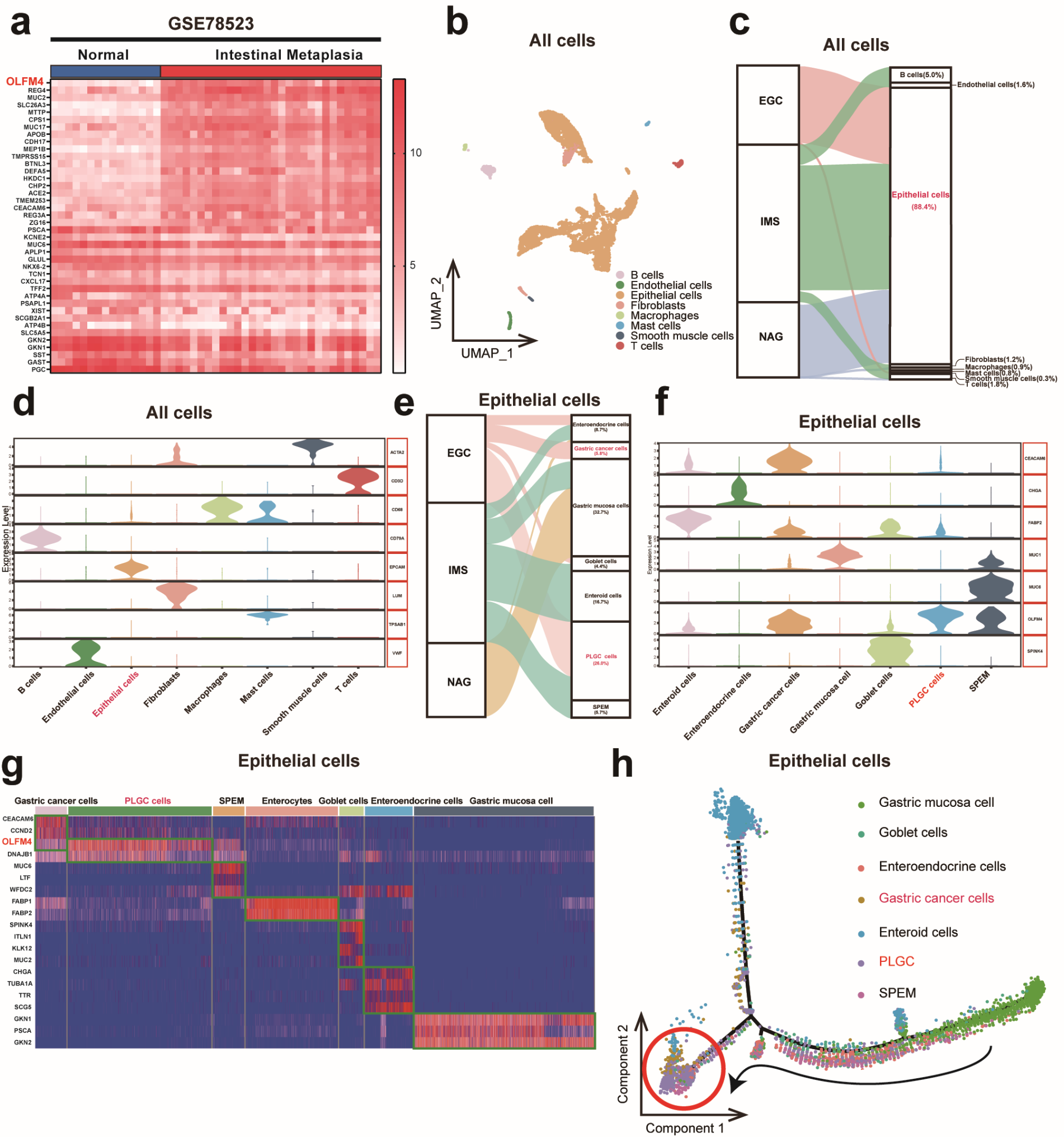


**Figure S1 OLFM4 was the biomarker of PLGC tissue:** (a) Heatmap displaying the top 20 DEGs in intestinal metaplasia, with OLFM4 ranking as the most highly expressed. (b-d) The cluster analysis, Sankey plot, violin plot showed the cell subgroup proportions and identification following the initial clustering of data from GSE134520. (e-g) The Sankey plot, violin plot and the heatmap showed the assessment of epithelial cell subpopulation proportions and identification of cell subpopulations through the second cluster analysis of GSE134520 data. (h) Monocle analysis depicting the differentiation trajectories of epithelial cell subgroups.


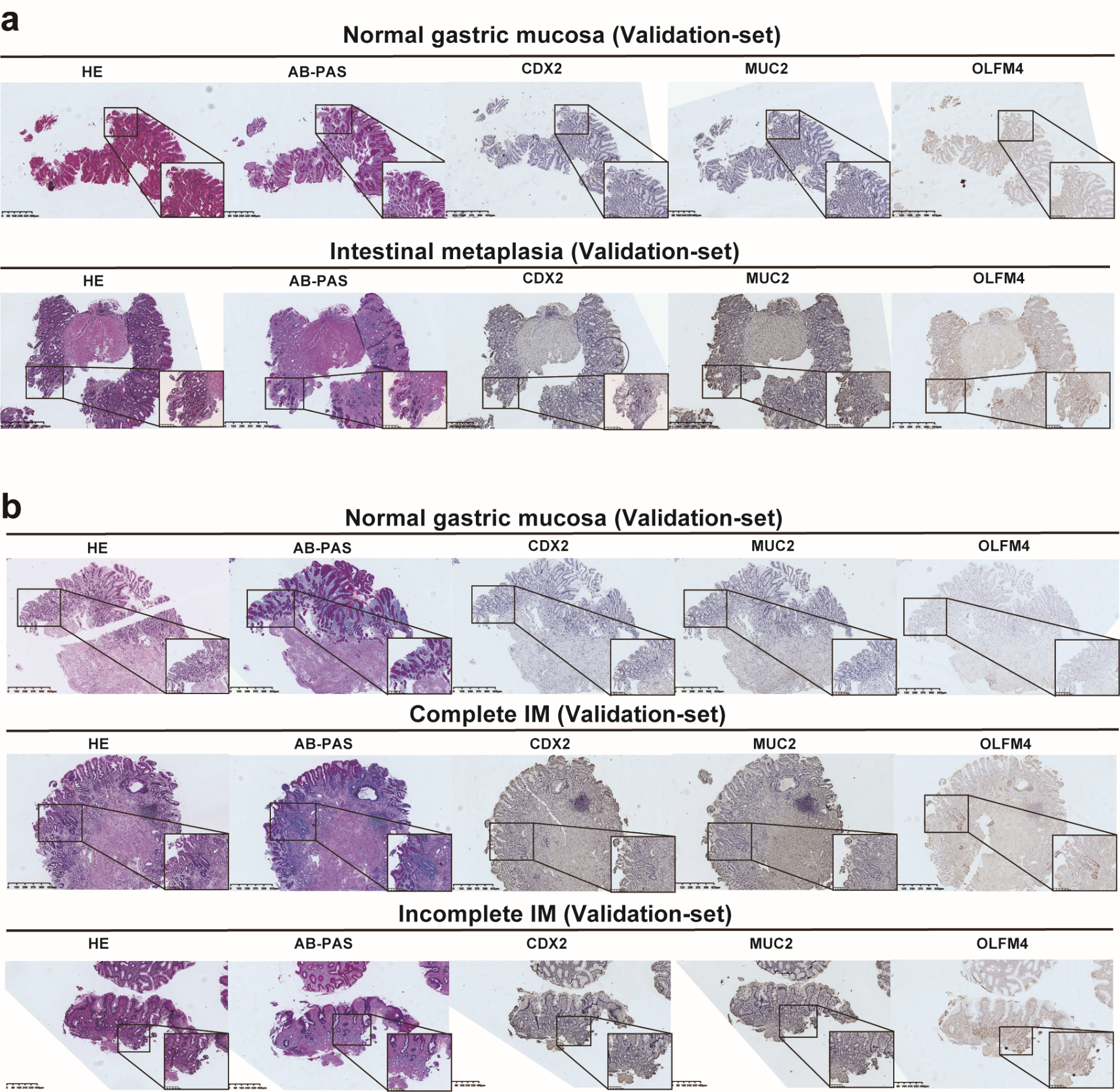


**Figure S2 OLFM4 was the biomarker of IIM tissue:** (a) HE staining and AB-PAS staining were employed to confirm the diagnosis of intestinal metaplasia and the immunohistochemical status was assessed by the expression of CDX2, MUC2, and OLFM4 in the Validation set. (b) HE staining and AB-PAS staining were utilized to confirm the diagnosis of intestinal metaplasia subtypes and the immunohistochemical status was evaluated by the expression of CDX2, MUC2, and OLFM4 in the Validation set.


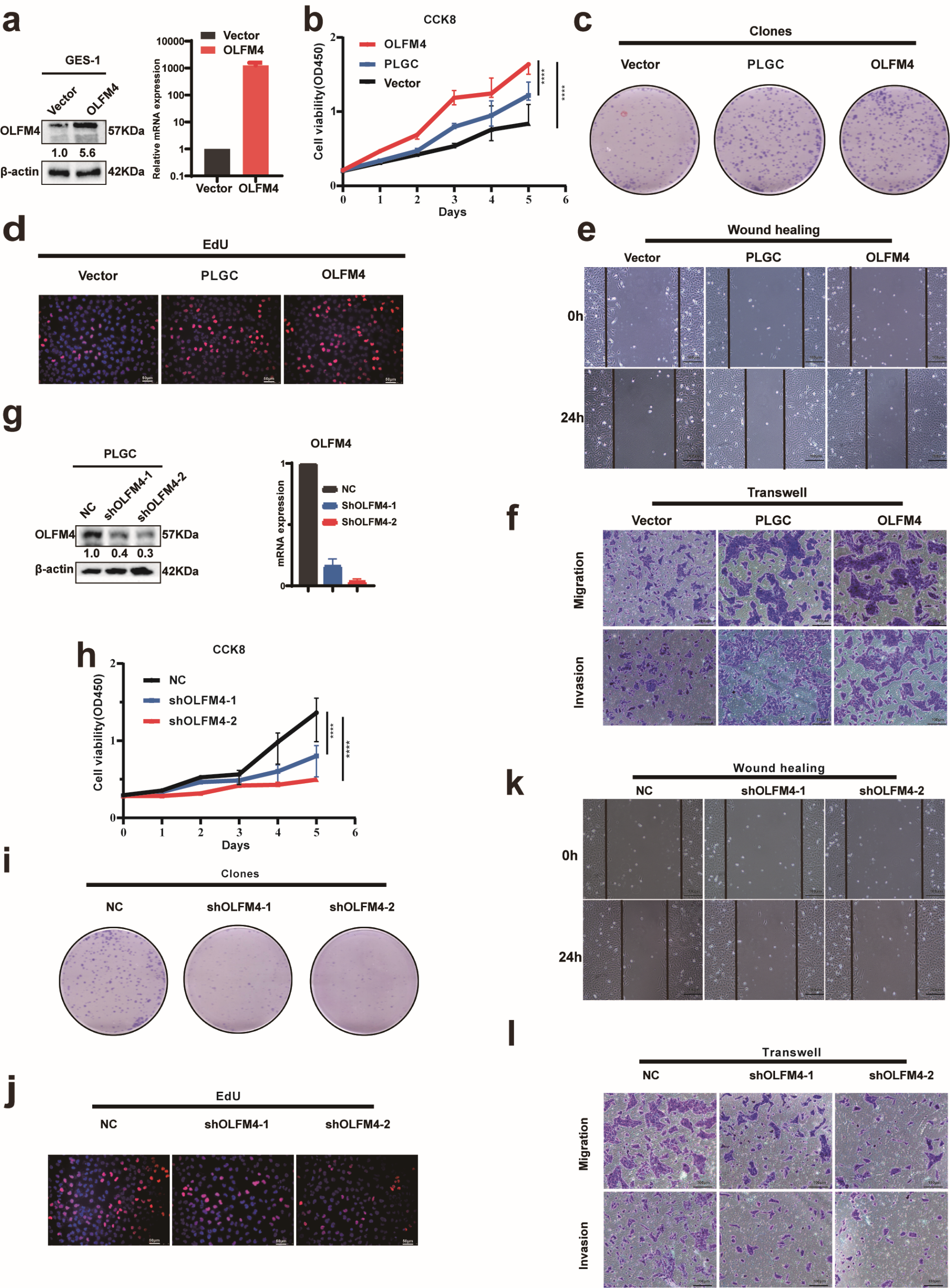


**Figure S3** **OLFM4 promoted the ability of proliferation and invasion:** (a) Western Blotting and qPCR confirmed increased OLFM4 expression in oeOLFM4 cells. (b-f) CCK8, cell cloning, EdU, wound healing, and transwell assays collectively demonstrated enhanced proliferation and invasion abilities in PLGC cells and oeOLFM4 cells. (g) Western Blot and qPCR confirmed decreased OLFM4 expression in shOLFM4 cells and the knockdown efficiency of OLFM4 was calculated by Image J or Graphpad prism. (h-l) CCK8, cell cloning, EdU, wound healing, and transwell assays collectively revealed weakened proliferation and invasion abilities in shOLFM4 cells.


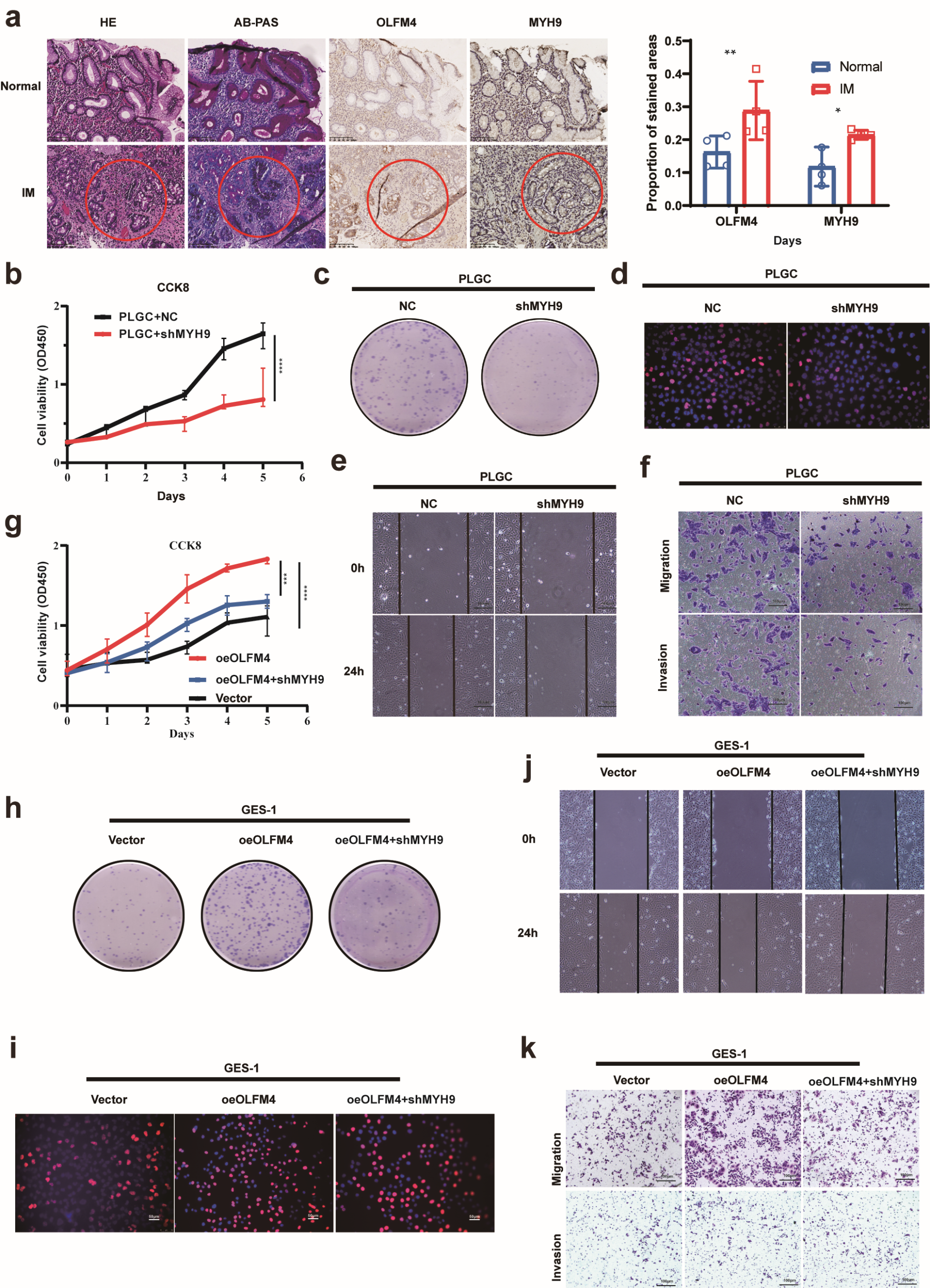


**Figure S4 OLFM4 interacted with MYH9 to promote proliferation and invasion** (a) HE staining and AB-PAS staining were employed to confirm the diagnosis of intestinal metaplasia and the immunohistochemical status was assessed by the expression of OLFM4 and MYH9 on pathological slides. The regions where intestinal metaplasia cells were present were indicated by red circles. (b-f) CCK8, cell cloning, EdU, wound healing, and transwell assays collectively demonstrated reduced proliferation and invasion capabilities in shMYH9 cells. (g-k) CCK8, cell cloning, EdU, wound healing, and transwell assays collectively revealed weakened proliferation and invasion in oeOLFM4 cells treated with MYH9 knowdown.


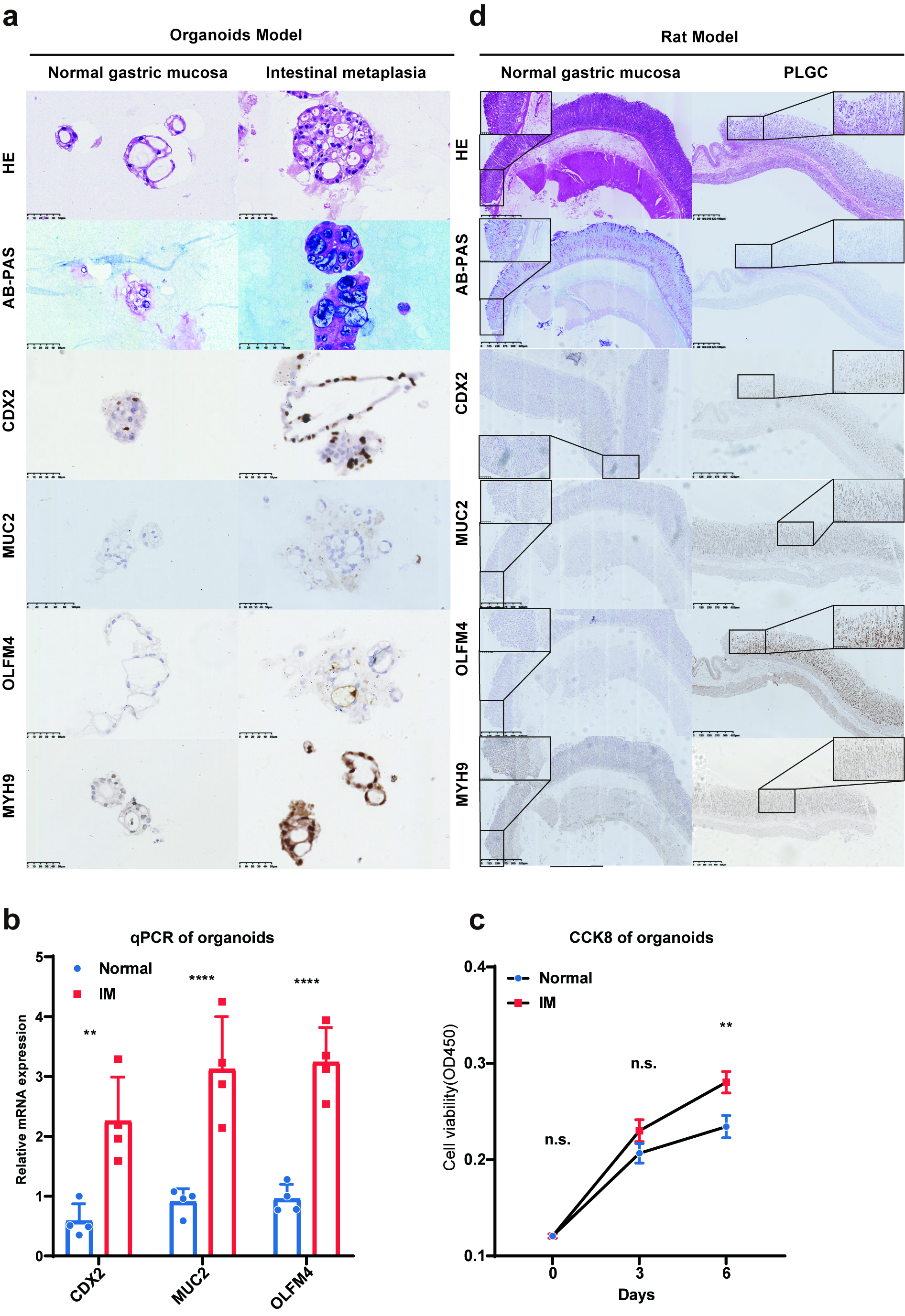


**Figure S5 OLFM4 and MYH9 expressed highly in IM tissues:** (a) HE staining, AB-PAS staining, and immunohistochemical expression of CDX2, MUC2, OLFM4, and MYH9 in intestinal metaplasia organoids. (b) qPCR was performed to access the relative mRNA expression of CDX2, MUC2, OLFM4 in normal gastric organoids or intestinal metaplasia organoids. (c) CCK8 accessed the cellular proliferation ability of normal gastric organoids or intestinal metaplasia organoids in 0, 3, 6 days. (d) HE staining, AB-PAS staining and immunohistochemical expression of CDX2, MUC2, OLFM4 and MYH9 in PLGC animal models.

**Supplemental table 1**

**The prediction on the impact of a variant on the OLFM4 protein tertiary structure**

| UniProt ID | Gene name | UniProt Position | Residue Wildtype | Residue Mutant | Structural damage predicted | Polyphen prediction score |
| --- | --- | --- | --- | --- | --- | --- |
| Q6UX06 | OLFM4 | 274 | TRP | LEU | Buried H-bond breakage | probably_damaging (1.00) |
| Q6UX06 | OLFM4 | 275 | GLY | ASP | Cavity altered | probably_damaging (1.00) |
| Q6UX06 | OLFM4 | 275 | GLY | ARG | Cavity altered; Buried / exposed switch | probably_damaging (1.00) |
| Q6UX06 | OLFM4 | 339 | TYR | HIS | Buried charge introduced | probably_damaging (1.00) |
| Q6UX06 | OLFM4 | 388 | ASP | GLY | Buried charge replaced; Buried H-bond breakage | probably_damaging (1.00) |
| Q6UX06 | OLFM4 | 393 | TRP | CYS | Cavity altered | probably_damaging (1.00) |
| Q6UX06 | OLFM4 | 406 | VAL | GLU | Buried hydrophilic introduced; Buried charge introduced | probably_damaging (1.00) |
| Q6UX06 | OLFM4 | 438 | GLY | ARG | Buried Gly replaced; Buried / exposed switch | probably_damaging (1.00) |
| Q6UX06 | OLFM4 | 440 | LEU | PRO | Buried Pro introduced | probably_damaging (1.00) |
| Q6UX06 | OLFM4 | 441 | TYR | HIS | Buried charge introduced; Buried H-bond breakage | probably_damaging (1.00) |
| Q6UX06 | OLFM4 | 441 | TYR | SER | Buried H-bond breakage | probably_damaging (1.00) |
